# Soybean photosynthetic and biomass responses to carbon dioxide concentrations ranging from pre-industrial to the distant future

**DOI:** 10.1093/jxb/eraa133

**Published:** 2020-03-12

**Authors:** David W Drag, Rebecca Slattery, Matthew Siebers, Evan H DeLucia, Donald R Ort, Carl J Bernacchi

**Affiliations:** 1 Department of Plant Biology, University of Illinois at Urbana-Champaign, Urbana, IL, USA; 2 Carl R. Woese Institute for Genomic Biology, University of Illinois at Urbana-Champaign, Urbana, IL, USA; 3 Institute for Sustainability, Energy, and Environment, University of Illinois at Urbana-Champaign, IL, USA; 4 Global Change and Photosynthesis Research Unit, United States Department of Agriculture, Agricultural Research Service, Urbana, IL, USA; 5 University of Essex, UK

**Keywords:** Biomass, elevated CO_2_, photosynthesis, soybean (*Glycine max*)

## Abstract

Increasing atmospheric carbon dioxide concentration ([CO_2_]) directly impacts C_3_ plant photosynthesis and productivity, and the rate at which [CO_2_] is increasing is greater than initially predicted by worst-case scenario climate models. Thus, it is increasingly important to assess the physiological responses of C_3_ plants, especially those that serve as important crops, to [CO_2_] beyond the mid-range levels used in traditional experiments. Here, we grew the C_3_ crop soybean (*Glycine max*) at eight different [CO_2_] levels spanning subambient (340 ppm) to the highest level thought plausible (~2000 ppm) in chambers for 5 weeks. Physiological development was delayed and plant height and total leaf area increased at [CO_2_] levels higher than ambient conditions, with very little difference in these parameters among the elevated [CO_2_] treatments >900 ppm. Daily photosynthesis initially increased with rising [CO_2_] but began to level off at ~1000 ppm CO_2_. Similar results occurred in biomass accumulation. Thus, as [CO_2_] continues to match or exceed the worst-case emission scenarios, these results indicate that carbon gain, growth, and potentially yield increases will diminish, thereby ultimately constraining the positive impact that continuing increases in atmospheric [CO_2_] could have on crop productivity and global terrestrial carbon sinks.

## Teaser

Increases in soybean photosynthesis and biomass diminish at growth CO_2_ concentrations >1000 ppm, constraining the benefits of increasing atmospheric CO_2_ concentration on soybean productivity.

## Introduction

The worst-case scenarios (IPCC RCP8.5 prediction) of future global carbon emissions are being exceeded by actual emissions (Jackson *et al.*, 2017; Le Quéré *et al.*, 2018). Since the advent of the Industrial Revolution, [CO_2_] has increased from below ~260 µmol CO_2_ mol^–1^ air (hereafter presented in parts per million, ppm; Raynaud and Barnola, 1985) to concentrations >400 ppm (Jones, 2013), which is unprecedented in the last 650 000 years and perhaps much longer (Zhang *et al.*, 2013). The majority of elevated [CO_2_] experiments, particularly chamber studies and those using free air concentration enrichment (FACE) technology, have focused on IPCC mid-range emission scenarios that suggest a doubling of [CO_2_] by the end of the century (Ainsworth and Long, 2005). If carbon emissions continue at their current rate of increase, these mid-range levels will be surpassed earlier than expected. Therefore, it is essential to better understand the effects of a higher range of [CO_2_] on plant growth and productivity.

The typical response of C_3_ photosynthesis to [CO_2_] supply {usually determined from photosynthetic [CO_2_] response (*A*/*C*_i_) curves} becomes insensitive to increases in atmospheric [CO_2_] above ~1000 ppm (Canadell *et al.*, 2007). A plateau in the [CO_2_] fertilization effect on photosynthesis will probably lead to a cessation in increases in plant biomass accumulation at higher [CO_2_]. The implications of this response are profound, as the terrestrial carbon sink is determined by net primary productivity, the majority of which occurs through C_3_ photosynthesis. If carbon assimilation through photosynthesis into biomass begins to level off, the terrestrial sink will also cease increasing at 1000 ppm [CO_2_], or possibly at lower levels due to other factors such as nutrients which are often limiting in natural systems.

To evaluate food security for the growing global population in the midst of rising [CO_2_], it is important to understand how CO_2_ concentrations beyond those typically studied in FACE experiments will affect major food crops. Most major crops utilize the C_3_ photosynthetic pathway, which is more responsive to increases in [CO_2_] than those using the C_4_ pathway (Ainsworth *et al.*, 2002; Bernacchi *et al.*, 2006; Leakey *et al.*, 2009; Kimball, 2016). Growth in elevated [CO_2_] generally increases C_3_ crop photosynthesis; however, photosynthetic acclimation can potentially reduce the increased stimulation of photosynthesis (Bernacchi *et al.*, 2005; Ainsworth and Rogers, 2007; Leakey *et al.*, 2009; Wang *et al.*, 2013; Bagley *et al.*, 2015; Kimball, 2016). Despite significant variation in acclimation responses (e.g. Ainsworth and Rogers, 2007) and acclimation not necessarily reducing photosynthetic potential (Bernacchi *et al.*, 2005), the extent to which photosynthetic acclimation may impact crop carbon uptake at [CO_2_] beyond the concentrations typically used in experiments, from ~500 ppm to 700 ppm, is relatively uncertain. The rapidly increasing rate of global anthropogenic CO_2_ emissions suggests that future [CO_2_], potentially exceeding 1200 ppm by the year 2100 (IPCC, 2014), may far exceed concentrations currently being used for experimentation, which necessitates an understanding of plant responses to much higher [CO_2_]. Few studies exist where growth [CO_2_] exceeds ~750 ppm. Early studies testing the effects of extreme levels of CO_2_ on plants have reported leaf necrosis and damage to other tissues with [CO_2_] enrichment to 1000 ppm (Peet *et al.*, 1986). This was thought to be a result of a greater amount of starch accumulation in the tissue (Madsen, 1975; Sasek *et al.*, 1985; Peet *et al.*, 1986) but could also have been the result of a contaminant in the exogenous CO_2_. Increased starch and carbohydrate accumulation in the leaf has also been hypothesized to trigger photosynthetic acclimation, where excess photosynthate has limited sinks to fill (Makino and Mae, 1999).

Soybean (*Glycine max*) is the most widely grown oilseed crop (Singh and Hymowitz, 1999), thus there are a large number of studies addressing the effects of increased [CO_2_] (~200 ppm greater than ambient) on its physiology, development, and productivity (Ainsworth *et al*., 2002, 2006; Bernacchi *et al.*, 2007; Leakey *et al.*, 2009; Twine *et al.*, 2013; Ruiz-Vera *et al.*, 2013; Rosenthal *et al.*, 2014; Gray *et al.*, 2016; Köhler *et al.*, 2017; Wang *et al.*, 2018; Li *et al.*, 2019). A meta-analysis covering 111 studies on soybean responses to elevated [CO_2_] ranging from 450 ppm to 1200 ppm found significant increases in light-saturated leaf photosynthetic rates, total canopy assimilation, growth rates, biomass, yield, branch number, leaf number, and stem heights between the control and elevated [CO_2_] conditions (Ainsworth *et al.*, 2002). However, this analysis was based on a compilation of published data and did not represent an analysis of these traits across a range of [CO_2_] within a controlled experiment. One previous chamber study in soybean showed increases in biomass up to 1000 ppm [CO_2_] but did not include higher levels (Allen *et al.*, 1991), whereas another examined physiology up to 1600 ppm [CO_2_] but with few treatment levels (five total), thereby limiting analyses of trends over [CO_2_] (Rogers *et al.*, 1983). Although a recent chamber study examined soybean leaf morphology across a range of [CO_2_] from 400 ppm to 1600 ppm and also reported photosynthetic rates, their findings showed very low rates of photosynthesis that declined with [CO_2_] up to 1200 ppm (Zheng *et al.*, 2019), which is contrary to theory as well as the majority of the literature on [CO_2_] fertilization effects. Thus, a more extensive examination of soybean photosynthesis including biomass data across an expansive [CO_2_] range is necessary for understanding future crop responses to [CO_2_].

In this study, we measured the response of soybean vegetative growth, development, photosynthesis, and photosynthetic physiology over a range of [CO_2_] from subambient concentrations to those exceeding the highest predictions for the end of this century. We hypothesized that soybean photosynthesis would increase linearly with [CO_2_] but reach a threshold above which increases in photosynthesis begin to diminish. We hypothesize that due to acclimation effects, the diminishing rate of photosynthesis would be greater than predicted from modelled output. We further predict that biomass accumulation would mimic the observed responses of photosynthesis. This prediction could have major implications for crop production and terrestrial CO_2_ sinks under future conditions.

## Materials and methods

### Growth conditions

Soybean (*Glycine max*) cv. Pioneer 93B15 seeds were sown in 30.5 cm diameter 15 liter round pots (Classic 2000) filled with a soilless medium (Sun Gro Sunshine LC1 Grower Mix; Sun Gro Horticulture, Agawam, MA, USA) in March 2013. Pots were supplemented with 57 g of 15-9-12 Osmocote Plus 3–4 month extended-release fertilizer (Everris, Geldermalsen, The Netherlands) 10 days after emergence (DAE). Plants were watered every other day throughout the duration of the experiment. The plants did not receive any pesticide applications, inoculations, or additional fertilizer, and did not show any signs or symptoms of diseases, pests, or nitrogen deficiency.

The experiment was conducted for 5 weeks in eight custom-built, clear-walled, aluminum-framed growth chambers (Supplementary Fig. S1 at *JXB* online) within a greenhouse room at the Plant Science Laboratory at the University of Illinois at Champaign-Urbana. Each of the eight chambers measured 1.4 m long by 0.9 m wide and 1.02 m tall. Chambers were covered in 0.2 mm thick clear Dura-Lar film (Grafix, Inc.) that allowed >88% transmission of natural sunlight while retaining [CO_2_] within the chamber (Supplementary Fig. S1). Natural light was supplemented using 1000 W metal halide lamps mounted 1.4 m above the chambers. The photoperiod was 14 h starting at 06.00 h and ending at 20.00 h local time. Photosynthetic photon flux density (PPFD; µmol m^–2^ s^–1^) was measured with Apogee SQ-110 quantum sensors at the top of the canopy within each chamber from 14 DAE to the end of the experiment. Temperature was controlled at 23 °C during the day and 17 °C at night.

A regression analysis approach was chosen to get the most power out of the experiment. Regression analysis allows for each chamber, each representing a different CO_2_ concentration, to act as an independent treatment. Thus, this study consisted of eight treatments which are analogous to statistical replicates (Cottingham *et al.*, 2005). Each of the chambers was assigned a different [CO_2_], which was rotated weekly among the eight chambers throughout the experiment to reduce the impact of any chamber bias. As the chamber concentrations were rotated, the plants assigned to each treatment were also rotated to ensure consistent [CO_2_] throughout the experiment. The plants were also systematically rotated clockwise as they were moved to different chambers to minimize border effects. CO_2_ was injected into each chamber independently through solenoid valves (Model ETO-3M-12VDC, Clippard Instrument Laboratory, Inc., Cincinnati, OH, USA). The [CO_2_] analyzers (SBA-4 or SBA-5; PP Systems, Inc., Amesbury, MA, USA) were accurate to <1% of the span concentration over the calibrated range for [CO_2_] (PP Systems, Inc.). The analyzers were calibrated before and 2 weeks into the experiment using a span gas providing 750 ppm CO_2_ (S.J. Smith Co., Urbana, IL, USA). For the chambers controlling [CO_2_] ≤1500 ppm, infrared gas analyzers (IRGAs) with an upper limit of 2000 ppm (SBA-4; PP Systems, Inc.) were used to determine [CO_2_] within the chamber. In the chambers with [CO_2_] >1500 ppm, IRGAs calibrated for 5000 ppm (SBA-5, PP-Systems Inc.) were deployed to avoid the instability of the SBA-4 analyzers near their upper limit. The two lowest treatments were achieved using custom-built CO_2_ scrubbers that circulated the chamber air through an enclosure filled with Sodasorb (W.R. Grace & Co., Chicago, IL, USA) to remove the higher ambient [CO_2_] in the greenhouse.

### Measurements

Plant height and developmental stage (as defined in Fehr and Caviness, 1977) were recorded three times weekly from emergence until harvest for each individual plant. Each of the developmental stages was assigned a number and averaged from the five plants per chamber. Gas exchange measurements were conducted twice during the experiment using a portable gas exchange system (LI-6400; LI-COR, Inc., Lincoln, NE, USA) calibrated according to the manufacturer’s specifications. The first set of measurements was conducted during early vegetative development (V1–V2) and the second during late vegetative/early reproductive (V6–R2) development. All measurements were conducted on the youngest, fully expanded leaf of the plant. The gas exchange measurements consisted of midday leaf photosynthesis, diurnal photosynthesis measurements, and photosynthetic [CO_2_] response (*A*/*C*_i_) curves. Midday gas exchange measurements were collected beginning at 13.00 h and concluded in ~2 h (all times are Central Daylight Savings Time) on three plants in each chamber on both measurement days. Solar noon at this location was 12.00±10 min between the gas measurement dates (based on www.esrl.noaa.gov/gmd/grad/solcalc). The block temperature of the gas exchange system was set to 23 °C for all measurements and the gas exchange systems reference [CO_2_] (CO2R) was set equal to the treatment level being measured. The global ambient [CO_2_] at the time of the experiment was 395.3 ppm. Diurnal measurements were collected from two plants in each treatment once every 2 h from 09.00 h until 18.00 h. Light in the gas exchange system was set to match the ambient PPFD measured in the room. From these measurements, the daily integral of carbon assimilation (*A*') was calculated for each plant and averaged for each [CO_2_]. *A*/*C*_i_ measurements were collected on two different plants during each set of measurements using an auto-program feature of the portable gas exchange system. Photosynthetic parameters were measured after stabilization at 13 different levels of [CO_2_] in the following order: 400, 300, 200, 100, 50, 400, 400, 700, 1000, 1300, 1900 and 2100 ppm. The data were then analyzed according to the method described previously (Long and Bernacchi, 2003) and adjusted for temperature (Bernacchi *et al*., 2001, 2003) using the PS-FIT software package (http://www.life.illinois.edu/bernacchi/links.html). This software uses the leaf model of photosynthesis (Farquhar *et al.*, 1980) to calculate the maximum rates of electron transport (*J*_max_) and maximum velocity of carboxylation by Rubisco (*V*_c,max_) from the measured responses.

Mitochondrial respiration was measured at night during the second measurement period using custom-built leaf cuvettes installed on the LI-6400 (Gillespie *et al.*, 2012), which measured respiration from entire trifoliate leaves. To correct respiration measurements for leaf area, each trifoliate was removed from the plant after measurement, and its leaf area was determined using a leaf area meter (LI-3000, LI-COR, Inc.)

Plants were harvested after 5 weeks. There was no clear evidence of root restrictions in the pots as the plants were harvested. At this time, two 1.9 cm leaf discs were sampled from the youngest most expanded leaf from three plants in each treatment and dried to constant mass to determine leaf mass per area (LMA). The number of leaves, nodes, and branches was counted and recorded for each of the five plants in each chamber. Stem height and basal circumference were also measured. Plants were then separated into leaves, stems, and roots. Leaf area was determined for each individual plant using a leaf area meter (LI-3000; LI-COR, Inc.). The separated organs of each plant were then oven-dried at 60 °C until constant mass was reached and weighed to determine individual dry weights, all of which were summed to determine total dry weight per plant.

Midday *A* was modeled based on the environmental conditions within the chamber for each CO_2_ treatment for each day using the leaf model of net carbon assimilation (Farquhar *et al.*, 1980) with temperature adjustments of key biochemical parameters (Bernacchi *et al*., 2001, 2003). Environmental conditions include leaf temperature, intercellular [CO_2_] (*C*_i_), and photosynthetically acitve radiation (PAR). Measured *V*_c,max_ and *J*_max_ from the treatment most closely resembling global mean [CO_2_] was used to model *A* for all treatments. For the second time period, the model parameterized with measured *V*_c,max_ and *J*_max_ overestimated *A* at all CO_2_ concentrations, thus requiring adjustments of the parameters to more accurately reflect measured *A* in the 400 ppm treatment.

### Statistical analysis

Plant height and developmental stage were averaged across the five plants within each treatment for each day of measurement. For developmental stages, averages were calculated by assigning a numerical value for each stage and calculating the mean from the five plants in each chamber on each measurement day. The relationships between [CO_2_] and height or gas exchange parameters were tested for statistical significance using regression analysis (Sigmaplot 12.5; Systat Software, Inc., San Jose, CA, USA). The shape of the responses of each parameter to [CO_2_] was used to determine the fit and type of regression used. Relationships between [CO_2_] and final harvest parameters were also tested using regression analyses on the averages of the five individual plants per treatment. Significance was determined at α=0.1.

## Results

### [CO_2_] treatments ranging from subambient levels to ~2000 ppm influence plant development and growth

**Fig. 1.**
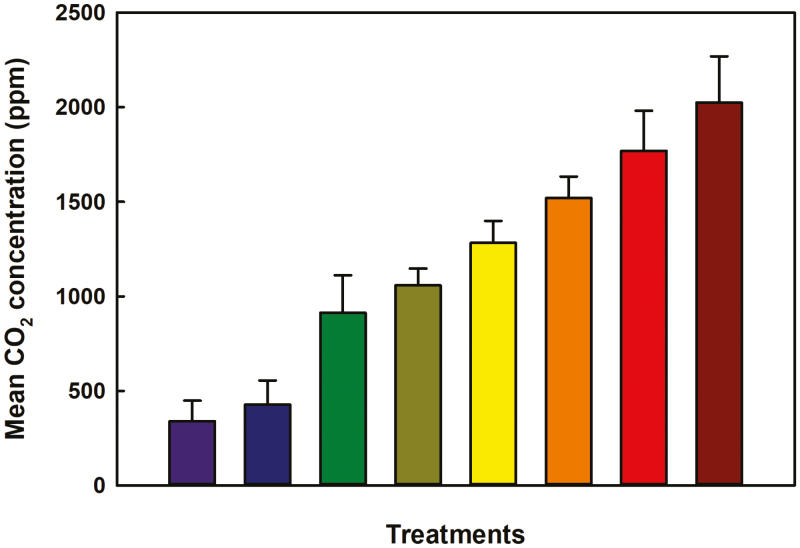
The mean [CO_2_] of each treatment over the entire duraion of the experiment calculated from 60 s of data. Error bars indicate 1 SD of the mean.

**Fig. 2.**
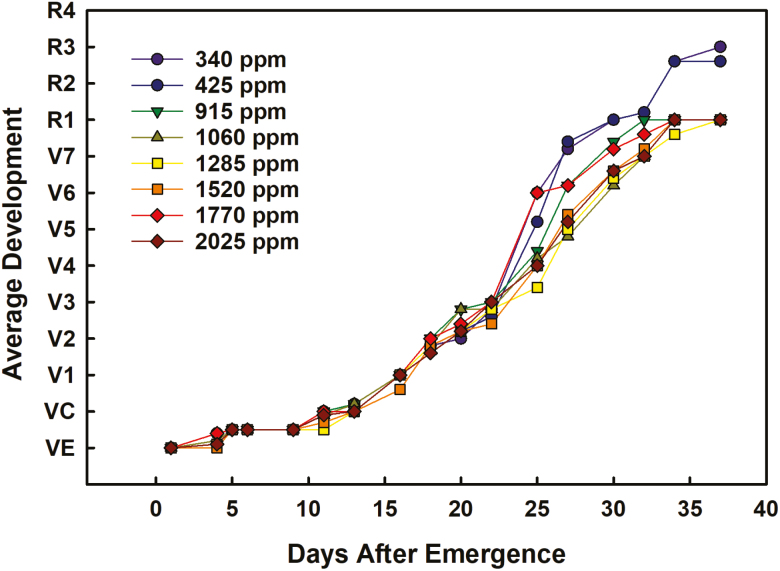
The progression through vegetative and reproductive development for soybean at each [CO_2_] treatment. The symbols represent the mean of five plants per chamber on each day.

**Fig. 3.**
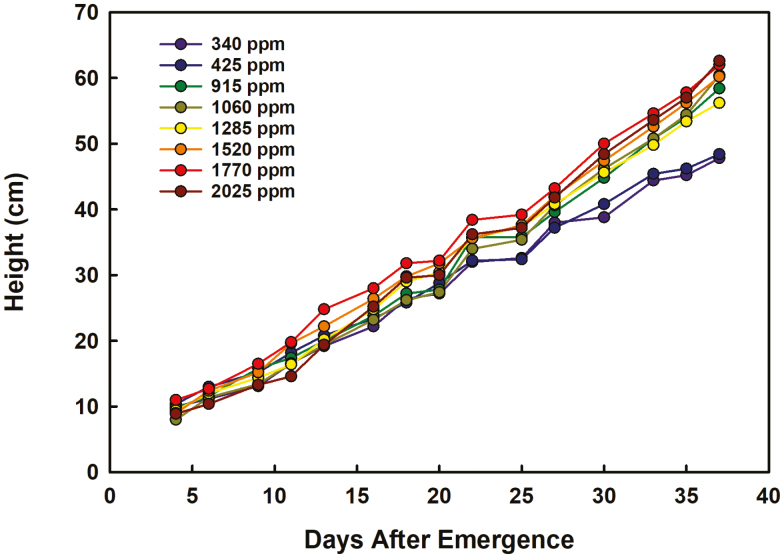
Plant height over the duration of the experiment. The symbols represent the mean of five plants per chamber on each day.

The average [CO_2_] within each treatment was calculated across the duration of the experiment, and the range of [CO_2_] spanned 340–2025 ppm (Fig. 1). The two lowest [CO_2_] treatments were slightly lower (~340 ppm) and slightly higher (425 ppm) than current ambient [CO_2_] (~400 ppm), whereas the elevated [CO_2_] treatments ranged from 915 ppm to 2025 ppm. Plant development lagged as [CO_2_] increased (Fig. 2). The difference in development among the treatments became apparent starting at 27 DAE, at which time plants in the two lowest [CO_2_] treatments were entering the sixth vegetative stage (V6), whereas those in the elevated [CO_2_] treatments were still in V3–V5. By 34 DAE, there was a clear separation between the two lowest [CO_2_] treatments and elevated [CO_2_] treatments, but there was little to no variation in developmental stage among the elevated [CO_2_] treatments (Fig. 2). Plant height increased with [CO_2_] (Fig. 3). A statistically significant relationship, either linear or hyperbolic, occurred among treatments at 16 DAE until the end of the experiment (Supplementary Fig. S2). By the end of the experiment, the difference in height from the lowest treatment to the highest was ~15 cm and, as with plant development, there was a clear separation between the two lowest [CO_2_] treatments and the elevated [CO_2_] treatments, with little variation among the elevated [CO_2_] treatment means (Fig. 3).

### [CO_2_] treatments affect leaf gas exchange parameters

**Fig. 4.**
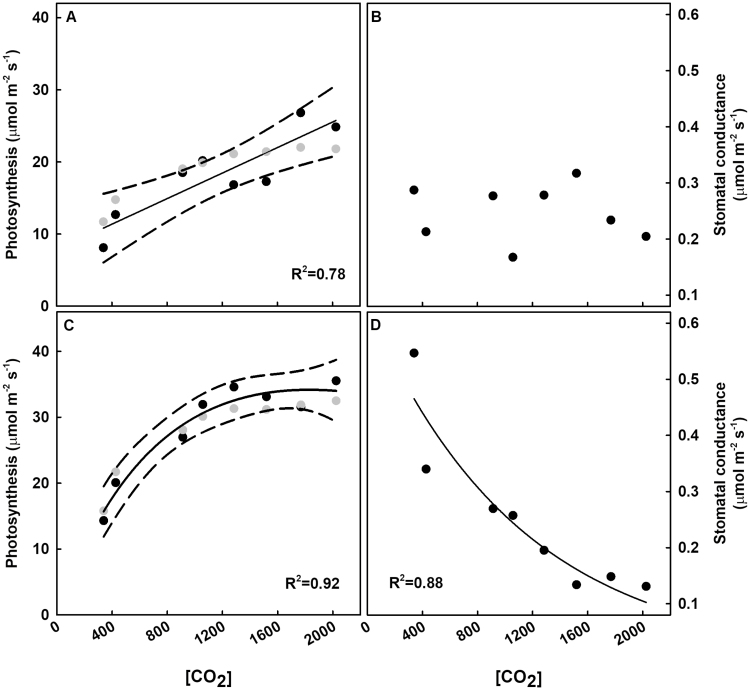
Midday soybean leaf gas exchange with [CO_2_]. Mean midday photosynthesis (A, C) and stomatal conductance (B, D) on the 16th (A, B) and 30th (C, D) day after emergence. Black symbols are the mean of three plants per treatment. Gray symbols (A and C) are modeled photosynthetic rates. Statistically significant relationships (*P*<0.1) as a function of [CO_2_] are indicated by a fit plotted through the data (solid line) and the 95% confidence limits (dashed line) for statistical comparison of modeled to measured photosynthetic rates. The *R*^2^ values are indicated in the figure.

**Fig. 5.**
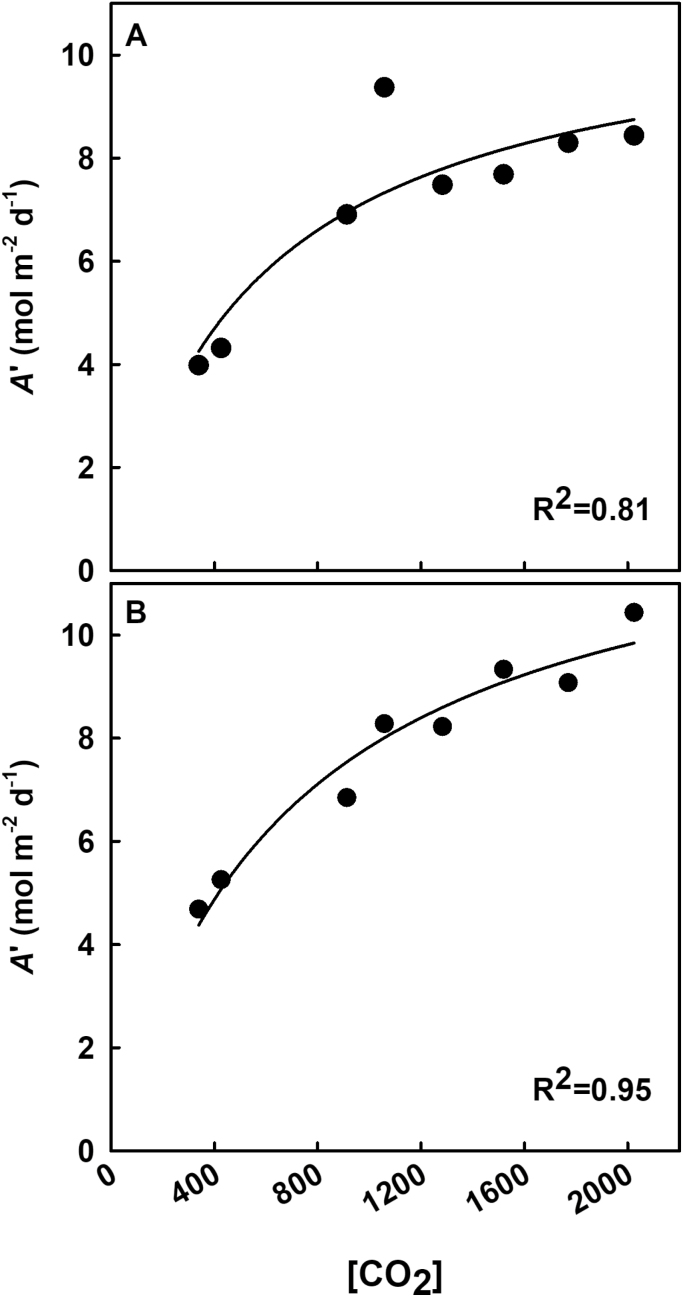
Integrals of daily photosynthesis as a function of [CO_2_]. Integrals (*A*') were calculated from diurnal measurements conducted on (A) 20 March (18 DAE) and (B) 3 April (32 DAE). Each point represents the average from each treatment. Statistically significant relationships (*P*<0.1) as a function of [CO_2_] are indicated by a fit plotted through the data. *R*^2^ values are indicated in the figure.

During the first measurement period (early vegetative at 16 DAE), midday leaf photosynthesis increased linearly with [CO_2_] (Fig. 4A). During the second measurement period (late vegetative/early reproductive at 30 DAE), midday leaf photosynthesis initially increased with [CO_2_] but began to plateau near 1000 ppm [CO_2_] (Fig. 4C). The relationship between [CO_2_] and stomatal conductance was not significant at 16 DAE (Fig. 4B), but there was a significant negative relationship (*P*<0.001) at 30 DAE (Fig. 4D) that began to recede at ~1000 ppm (Fig. 4D). Measured *A* did not deviate from modeled *A* for either measurement day, as indicated by the modeled values falling within the 95% confidence limits of the measured data (Fig. 4A, C). Although midday leaf photosynthesis increased linearly with [CO_2_] during the first measurement period, daily integrals of leaf photosynthesis increased more rapidly with [CO_2_] at low [CO_2_] than at high [CO_2_] (Fig. 5). On both days, the daily integrals of photosynthesis began to level off at ~1000 ppm CO_2_. The trend was more pronounced during the second set of measurements, as indicated by the higher *R*^2^ value.

**Fig. 6.**
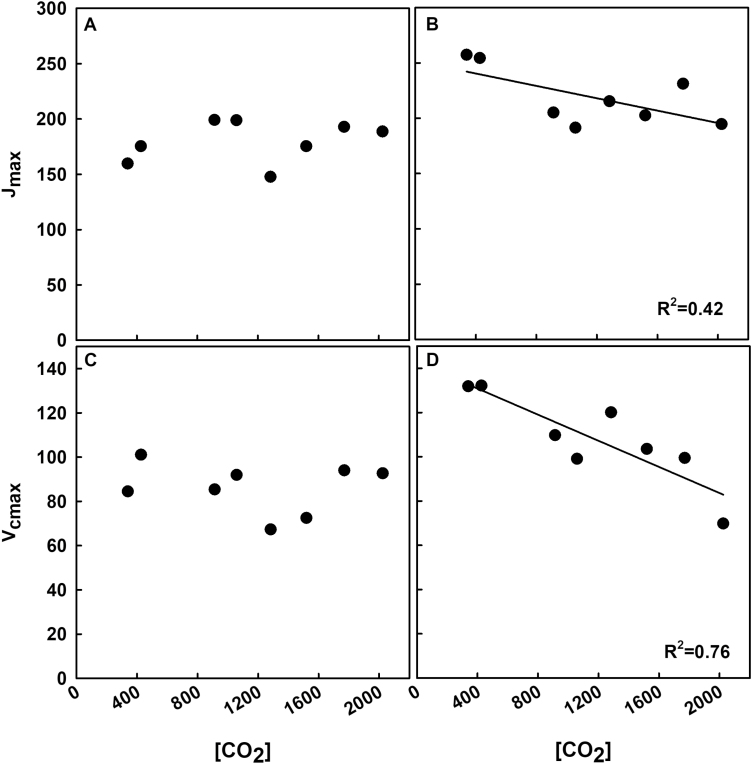
*V*_c,max_ and *J*_max_ values as a function of [CO_2_]. Values were calculated from *A*/*C*_i_ measurements conducted on (A, C) 21 March (19 DAE) and (B, D) 5 April (34 DAE) using the PS-Fit model. Each point represents the average from each treatment. Statistically significant relationships (*P*<0.1) as a function of [CO_2_] are indicated by a fit plotted through the data. *R*^2^ values are indicated in the figure.

**Fig. 7.**
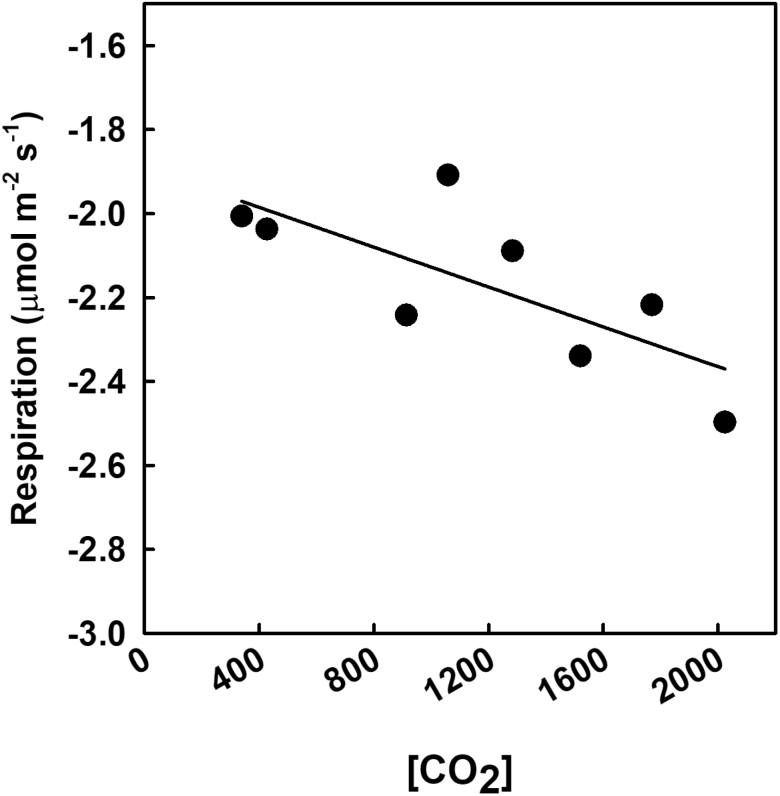
Dark respiration as a function of [CO_2_] measured at 35 DAE. Each point represents the average from each treatment. A statistically significant relationship (*P*<0.1) as a function of [CO_2_] is indicated by a fit plotted through the data. The *R*^2^ value is indicated in the figure.

The relationships between [CO_2_] and maximum rates of Rubisco carboxylation (*V*_c,max_) and electron transport (*J*_max_) determined from *A*/*C*_i_ curves were not statistically significant on the first day of measurements (19 DAE) (Fig. 6A, C). However, on the second day of measurements (35 DAE), *V*_c,max_ and *J*_max_ significantly decreased with increasing [CO_2_] (Fig. 6B, D). Leaf respiration was only measured late in the experiment (36 DAE). There was a significant negative trend in respiration with increasing [CO_2_] (Fig. 7).

### Growth [CO_2_] affects biomass accumulation and plant traits

**Fig. 8.**
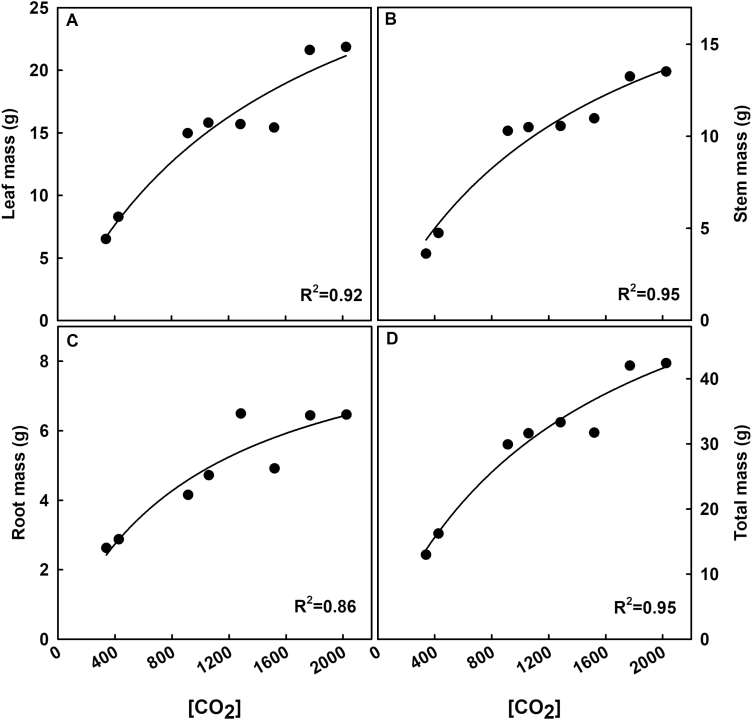
Dry biomass as a function of growth [CO_2_]. Biomass from leaves (A), stems (B), roots (C), and total plants (D) was dried and averaged within each treatment. Statistically significant relationships (*P*<0.1) as a function of [CO_2_] are indicated by a fit plotted through the data. *R*^2^ values are indicated in the figure.

**Fig. 9.**
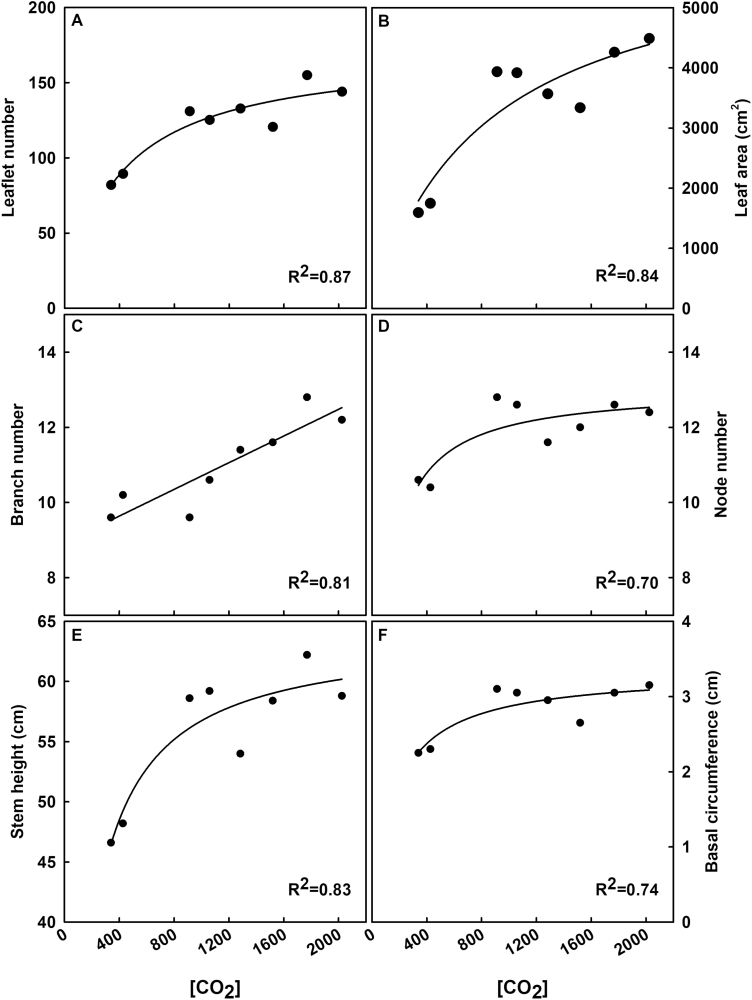
Plant traits from final harvest as a function of [CO_2_]. Parameters include mean (A) leaflet number, (B) leaf area, (C) branch number, (D) node number, (E) stem height, and (F) basal stem circumference. Each point represents the plant-level average from each treatment. Statistically significant relationships (*P*<0.1) as a function of [CO_2_] are indicated by a fit plotted through the data. *R*^2^ values are indicated in the figure.

All final harvest parameters collected increased steeply with [CO_2_] at concentrations <900 ppm but largely lessened at concentrations of ≥900 ppm (Fig. 8). Plant-level characteristics, such as basal stem circumference, leaf number, branch number, node number, leaf area, and stem height, all initially increased with [CO_2_] and then showed more subtle increases except for branch number (Fig. 9). LMA did not show any significant difference or trend among treatments (Supplementary Fig. S3).

## Discussion

The majority of studies examining the effects of elevated [CO_2_] on plants focus on [CO_2_] elevated between ~200 ppm and 350 ppm above ambient concentration, which is increasingly likely to be exceeded in the atmosphere in a relatively short number of decades. Thus, we studied the impact of [CO_2_] ranging from subambient levels to much higher levels than predicted for the end of this century on soybean growth and physiology. Similar to previous elevated [CO_2_] studies, we found that elevated [CO_2_] treatments delayed development and produced taller plants in the first 35 d of growth but with very little difference occurring among the elevated [CO_2_] treatments. Photosynthesis generally increased rapidly with [CO_2_] dosage below 900 ppm [CO_2_], but increases began to diminish at this point. Plant biomass initially increased with [CO_2_] dosage levels but, similar to photosynthesis, increases tailed off at ~900 ppm. Thus, increasing [CO_2_] leads to higher photosynthesis and growth with small increases in [CO_2_] in soybean, but the impact of [CO_2_] diminishes at progressively higher treatment levels, which may have large implications for food production and global carbon sink strength in future conditions. Contrary to our predictions, the measured increase in photosynthesis with rising [CO_2_] did not deviate substantially from modeled *A* parameterized to current atmospheric [CO_2_].

### Soybean development differs between near-ambient and elevated [CO_2_] treatments but not among elevated [CO_2_] levels

Developmental differences among the treatments occurred at 27 DAE (Fig. 2), at which time the elevated [CO_2_] treatments lagged the [CO_2_] treatments near ambient levels by 1–3 vegetative stages. By the end of the experiment, near-ambient [CO_2_] and elevated [CO_2_] treatments were clearly separated, with the near-ambient [CO_2_]-treated plants in full bloom (R2) and elevated [CO_2_]-treated plants mostly in late vegetative stage (V7), with little to no variation in developmental stage among the elevated [CO_2_] treatments. This delay is similar to that found in previous reports for soybean (Castro *et al.*, 2009) using the same soybean cultivar within a FACE experiment, where the initiation of early reproductive stages was most affected by [CO_2_] enrichment. In that experiment, the delay was probably caused by the formation of extra nodes on the plants under increased [CO_2_], and smaller, but significant, differences were also observed later in reproductive development (Castro *et al.*, 2009). By final harvest in this study, node number per plant also increased slightly with [CO_2_] dosage. The duration of the experiment was limited by chamber size, but a longer experiment would allow comparison of entry into reproductive stages among elevated [CO_2_] treatments and duration of key stages, such as grain filling, which is an important determinant of yield in soybean. Differences in plant height were statistically resolvable starting at 16 DAE (Supplementary Fig. S2), and plants in the highest [CO_2_] treatments were 15 cm taller, on average, than those in the lowest [CO_2_] treatments by the end of the experiment (Fig. 3). Similar differences in plant height were observed in control (390 ppm) and elevated (550 ppm) [CO_2_] FACE-grown soybean of the same cultivar over two growing seasons (Morgan *et al.*, 2005). As the soybeans in this study had not reached their full height, we cannot be sure if mature plant height would significantly differ among elevated [CO_2_] treatment levels. If so, this could have implications on lodging, which could lead to more severe reductions in harvested yields as [CO_2_] increases past mid-range levels.

### Soybean photosynthesis initially increases with [CO_2_] dosage but increases begin to diminish near 1000 ppm [CO_2_]

Overall, the observed differences in both midday and daily integrated photosynthesis between control and elevated [CO_2_] support the hypothesis that carbon assimilation will initially increase with rising [CO_2_] but diminish above a certain threshold (Figs 4, 5). It has been well documented that C_3_ species show increased photosynthetic assimilation with increases in [CO_2_] (Makino and Mae, 1999; Ainsworth *et al.*, 2002; Bernacchi *et al.*, 2005; Leakey *et al.*, 2009), but the elevated [CO_2_] treatments in these previous studies were lower than the highest levels used in this study. In a recent study, soybean photosynthesis decreased with [CO_2_] to a minimum at 1200 ppm (Zheng *et al.*, 2019), but the overall rates of photosynthesis were much lower than those of other soybean studies and of this study. Here, photosynthesis followed a hyperbolic trend, which trended toward steadily increasing until 900–1000 ppm where it began to plateau, suggesting saturation of photosynthetic enzymes, such as Rubisco, or carbon sinks. Interestingly, stomatal conductance on the first measurement day showed no differences among treatments, whereas the second measurement period showed the anticipated strong inverse relationship between stomatal conductance and [CO_2_]. This may have been a result of meteorological conditions, particularly overcast conditions with a PPFD of ~20% of maximum (~400 μmol m^–2^ s^–1^) during the first measurement date. The same measurement date also showed photosynthetic rates that were lower than those of the second measurement date, particularly at the lower [CO_2_] (Fig. 4).

Measured and modeled photosynthesis generally followed the same pattern, suggesting that the role of acclimation of *V*_c,max_ and *J*_max_ in midday photosynthetic rates may not decrease photosynthetic potential (Fig. 4A, C). A similar result was observed for soybean grown at elevated CO_2_ at the SoyFACE facility (Bernacchi *et al.*, 2005) and modeled for soybean at the canopy scale (Bagley *et al.*, 2015). The results here, however, suggest that the lack of influence of acclimation extends beyond the ~200 ppm increase in CO_2_ of these previous studies.

### Photosynthetic capacity parameters *V*_c,max_ and *J*_max_ decrease with [CO_2_] dosage during late vegetative/early reproductive stages

The first sampling in early vegetative development did not show a significant difference in *V*_c,max_ or *J*_max_ across treatments, but the second measurement period during late vegetative/early reproductive growth revealed a decline in *V*_c,max_ and *J*_max_ with rising [CO_2_] (Fig. 6). Previous studies have shown that pot size, chamber conditions, and nitrogen availability can strongly influence photosynthetic responses to elevated [CO_2_] (Sage, 1994; Ainsworth *et al.*, 2002; Bernacchi *et al.*, 2005). During the final harvest when roots were separated and washed for biomass, it was very apparent that the roots were not pot bound. There was still plenty of room for the roots to expand with no roots circling the soil mass, and the plants did not show any sign of stunted growth. The use of the Osmocote extended-release fertilizer provides sufficient nitrogen for 3–4 months, which surpassed the longevity of the experiment. The temperature set point, frequency of watering, and growing media all helped to extend the release of nutrients from the fertilizer. Studies looking into this found that polymer controlled-release fertilizers, such as Osmocote, will rarely release all of their nutrients in the time period labeled (Adams, 2010; Broschat, 2005). No signs or symptoms of nutrient deficiency were apparent. Moreover, these plants were beginning reproductive stages so had a strong carbon sink. Down-regulation of *V*_c,max_ often occurs in field experiments at elevated [CO_2_] (Ainsworth and Long, 2005), so we think it unlikely that these results are due to the plants being potted.

### The benefits of [CO_2_] on biomass accumulation diminish at [CO_2_] greater than 900 ppm

Destructive harvest results support the hypothesis that, during continuing atmospheric change, biomass accumulation will initially increase with rising [CO_2_] but lessen as [CO_2_] increases over 900 ppm. The final root, stem, leaf, and total masses revealed a linear trend toward higher biomass with rising [CO_2_] below 900 ppm (Fig. 8). The increases in photosynthesis at these levels probably provided more photosynthate that corresponded to the increases in biomass accumulation. As [CO_2_] surpassed 900 ppm, increases in biomass diminished, although a slight increase was observed at the highest [CO_2_] levels. This plateau in biomass mirrored the plateau in photosynthesis, suggesting that the limitation to biomass was carbon based. Although respiration tends to increase with increased [CO_2_] (Leakey *et al.*, 2009), we saw a decrease in respiration rates as [CO_2_] increased (Fig. 7). Although we cannot be sure why, this suggests that respiration was not limiting carbon gain in this experiment.

### The diminishing effects of [CO_2_] at progressively higher treatment levels may impact future food production and global carbon sink strength

Soybean is an important C_3_ legume crop and is grown on >120 Mha globally (FAO, 2019). Although we were unable to extend the experiment to measure yields and despite the experiment not representing true plant canopies, the cessation of increases in photosynthesis and biomass seen in this study suggests that the productivity of soybean and probably other C_3_ crops will show a similar response as [CO_2_] reaches levels of 900–1000 ppm or higher. This could have major implications for food productivity in the midst of a growing human population and other facets of climate change, such as rising temperatures. Moreover, much of terrestrial primary productivity comes from C_3_ plants. Thus, the tailing off of increases in both photosynthesis and biomass of the C_3_ plant used in this study, which is similar to the saturation in typical *A*/*C*_i_ curves of C_3_ plants (Canadell *et al.*, 2007), suggests limitations to the global carbon sink as growth [CO_2_] reaches levels of 1000 ppm. However, the level of [CO_2_] at which the plateau may occur could vary substantially based on many factors including, but not limited to, interspecific variation, plant functional types, nutrient availability, and global changes. This has major implications, as the terrestrial sink is currently responsible for mitigating increases in atmospheric [CO_2_] (Keenan *et al.*, 2016), and [CO_2_] levels could increase at even faster rates if the terrestrial sink becomes limited in high [CO_2_] conditions.

### Conclusions

In this study, we subjected soybean plants to a range of [CO_2_] values intended to represent the uncertainty related to future global CO_2_ emissions, which may result in rates that far exceed the perceived worst-case scenario. Our results show that rising [CO_2_] has a diminishing impact on photosynthesis and productivity above 1000 ppm during early stages of soybean growth. If these trends continue in later stages as we expect they will and translate to diminished returns of [CO_2_] on yield, strategies for overcoming these limitations, such as genetic engineering, will be required to realize potential benefits of elevated [CO_2_] on C_3_ crop yield. However, as these approaches are not feasible in natural systems, global terrestrial sinks may become limited and unable to mitigate further increases in [CO_2_].

## Supplementary data

Supplementary data are available at *JXB* online.

Fig. S1. A representative clear-sided chamber and the distribution of the five plants within the chamber inside a greenhouse.

Fig. S2. Plant height as a function of [CO2] during each day of measurement.

Fig. S3. Leaf mass per area (LMA) as a function of [CO2].

eraa133_suppl_Supplementary_Figure_S1_S3
