# Supplementary material for: Soybean photosynthetic and biomass responses to carbon dioxide concentrations ranging from pre-industrial to the distant future
Source: J Exp Bot. 2020 Mar 12;71(12):3690–700. doi: 10.1093/jxb/eraa133 (PMC7475242; doi:10.1093/jxb/eraa133)
Supplement: eraa133_suppl_Supplementary_Figure_S1_S3 [file eraa133_suppl_supplementary_figure_s1_s3.pdf]

**Supplemental Figures for: Soybean Photosynthetic and Biomass Responses to Carbon Dioxide Concentrations Ranging from Pre-Industrial to the Distant Future**

**David W. Drag[1,2], Rebecca Slattery[2], Matthew Siebers[1], Evan H. DeLucia[1,2,3], Donald R. Ort[1,2], Carl J. Bernacchi[1,2,3,4]***

[1] Department of Plant Biology, University of Illinois at Urbana-Champaign, Urbana, Illinois, United States.

[2] Carl R. Woese Institute for Genomic Biology, University of Illinois at Urbana-Champaign, Urbana, Illinois, United States.

[3]Institute for Sustainability, Energy, and Environment, University of Illinois at Urbana-Champaign, Illinois, United States.

[4]Global Change and Photosynthesis Research Unit, United States Department of Agriculture, Agricultural Research Service, Urbana, Illinois, United States

**Corresponding author**: Carl J. Bernacchi (carl.bernacchi@usda.gov)

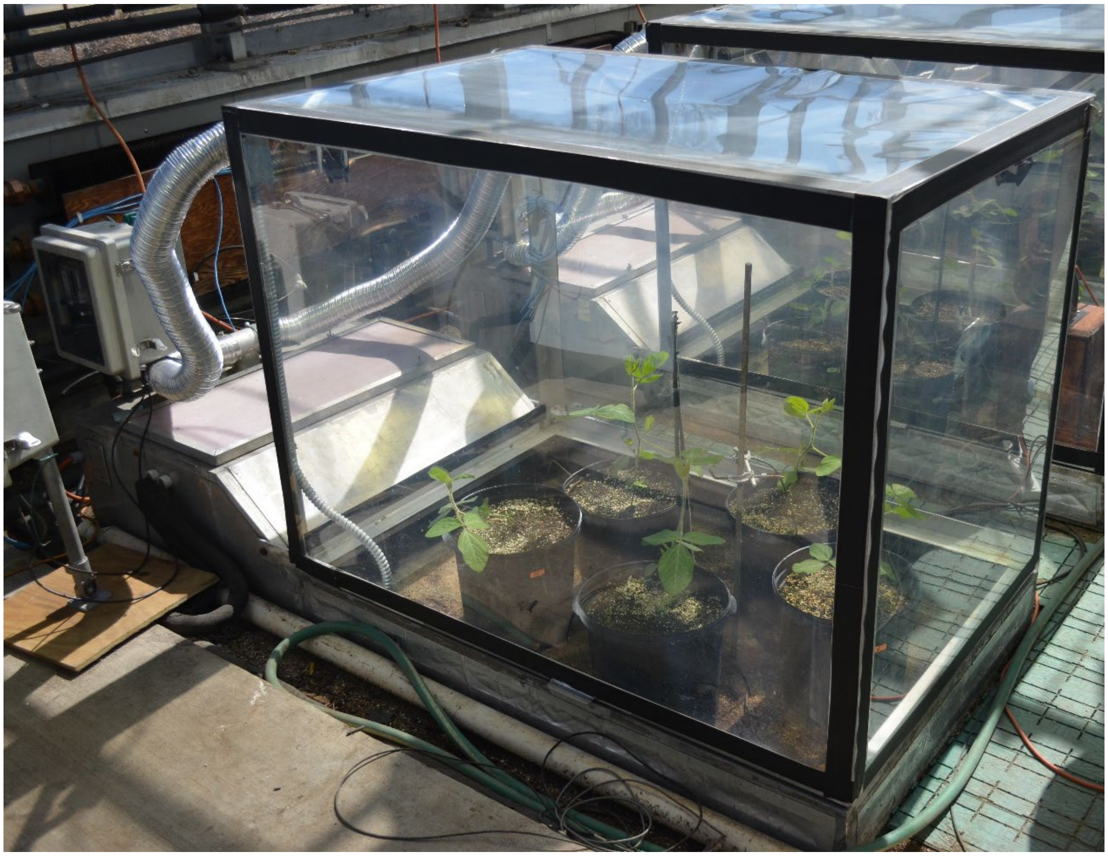

Supplemental Figure S1. Representative chamber. Photograph shows a representative clear-sided chamber and the distribution of the five plants within the chamber inside a greenhouse.

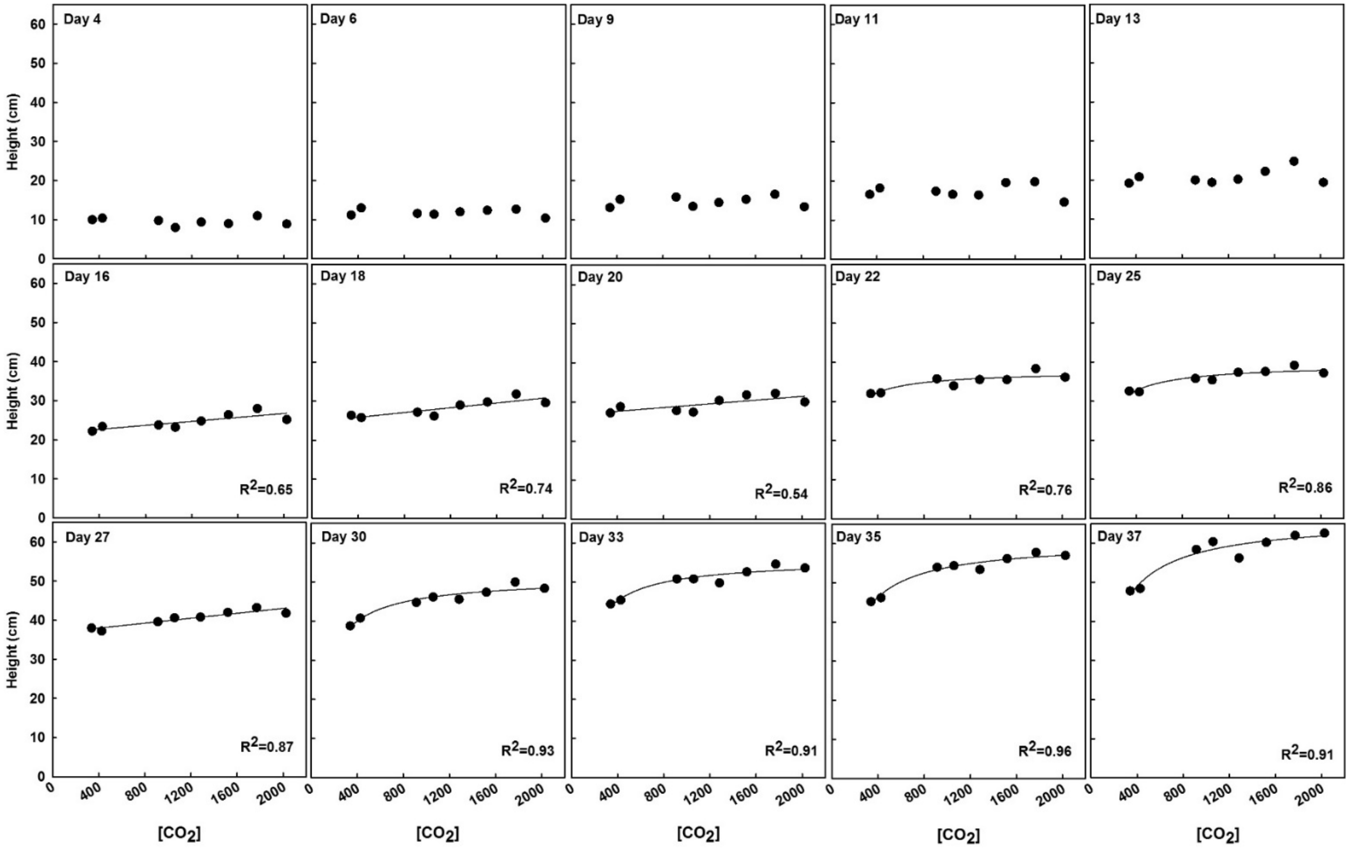


Supplemental Figure S2. Plant height as a function of [$CO_2$] within each day of measurement. Day of measurement is indicated in the upper left-hand corner of each panel and corresponds to the days of measurement shown in Figure 3. Statistically significant relationships ($p$<0.1) as a function of [$CO_2$] are indicated by a fit plotted through the data. $R^2$ values are indicated in the figure.

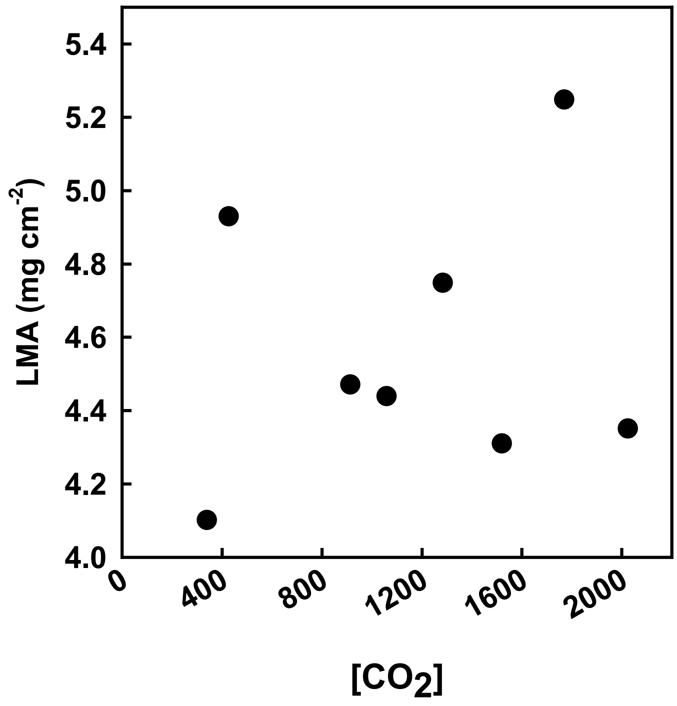


Supplemental Figure S3. Leaf Mass per Area (LMA) as a function of [$CO_2$]. Values are the means of the five plants within each treatment. There was no significant relationship among treatments.
